# Power, Process and Context in Theory Based Evaluation of Policy Implementation: A Response to Recent Commentaries

**DOI:** 10.15171/ijhpm.2018.109

**Published:** 2018-11-11

**Authors:** Angela Lawless, Fran Baum, Toni Delany, Colin MacDougall, Carmel Williams, Dennis McDermott, Helen van Eyk

**Affiliations:** ^1^College of Nursing and Health Sciences, Flinders University, Adelaide, SA, Australia.; ^2^Southgate Institute for Health Society and Equity, Flinders University, Adelaide, SA, Australia.; ^3^Discipline of Public Health, Flinders University, Adelaide, SA, Australia.; ^4^SA Health, Department of Health and Wellbeing, Adelaide, SA, Australia.; ^5^The Poche Centre for Indigenous Health and Wellbeing, Flinders University, Adelaide, SA, Australia.

Our paper^1^ describes the development of an evaluation framework using program theory. Our research examined the effectiveness of program logic and action research to determine the impact of a complex multi-sectoral policy initiative on population health. The commentaries argue that this approach, with some caveats, holds promise as a foundation for future Health in All Policies (HiAP) research. Shankardass et al^2^ note the potential of adapting the approach to produce bespoke frameworks for other contexts. Labonté^3^ suggests that the approach provides a useful ‘roadmap’ combining methodological rigour, practice and theory. Holt and Ahlmark^4^ note the potential for a better understanding of HiAP mechanisms. We found this approach allowed us to navigate the complexity of policy-making and articulate the pathways through which policy brings desired outcomes.

Labonté^3^ notes that our team has a strong commitment to working with stakeholders and we believe this made the research feasible. The early involvement of policy makers highlighted by Harris^5^ facilitated data gathering and dissemination of findings. de Leeuw^6^ raises caution about HiAP researchers “placing themselves in the same part of the field as the public (sector) health bureaucracy.” Her assertion that it may be “self-delusional” to assume shared ideals suggests researchers have not considered their positionality in the research or have no understanding of medical dominance and health system critiques. We argue that both are indeed pre-requisites for engaging in HiAP research and our track record demonstrates our awareness of these.

A number of suggestions were made for strengthening or modifying our approach. Shankardass et al^2^ suggest that engagement could be further developed through adoption of a Developmental Evaluation approach.^7^ We agree and note that it has considerable overlap with action research. Holt and Ahlmark^4^ call for greater ‘specification of variables and causal mechanisms.’ The framework presented in our original article is an overarching model of HiAP in South Australia. Our application of the framework to specific case studies allowed for such greater specificity. There is also merit in their suggestion to focus on fewer causal relations. A staged exploration of the overarching framework may identify particular components and links for detailed secondary examination. Labonté^3^ proposes that more attention to macro level political factors is needed. We agree but also note that empirical work needs to have a realistic scope and focus.

de Leeuw^6^ and Harris^5^ call for more critical engagement with issues of power, politics and institutional context. The program theory does posit that implementation of the HiAP strategies is mediated by institutional factors, power relationships, political will and resources, however the observation by Peña^8^ that these factors are depicted in a way that visually undermines their importance is a constructive reflection. We were alert to power as a key factor shaping all components and links. We explored these issues and sought evidence about how power played out. de Leeuw^6^ calls for researchers to “take a look at the sources of power and their distribution among the particular configurations of stakeholders around the issues” taking the healthcare system and bureaucracies out of the question. We contend that program theory based evaluation enabled identification of those sources and configurations. Furthermore, we found that forces affecting the health system, such as neoliberalism and individualism, also influenced HiAP.

The use of multiple theories to interpret our results and test and develop program theory was seen as both a strength and limitation. Like Shankardass et al^2^ we believe future work would benefit from transdisciplinary approaches and integration of theories from diverse fields including political science and economics. Indeed we have further examined theory driven analysis in an in-depth engagement between public health and social science researchers, and health bureaucrats.^9^ This workshop reinforced the potential of multidisciplinary approaches and the value of using a range of theories.

We thank the commentators for recognising the potential of our HiAP evaluation model and hope others will learn from and develop our work.

## Acknowledgements

This work was supported by the National Health and Medical Research Council (grant number 1027561).

## Ethical issues

Not applicable.

## Competing interests

CW is the Manager, Health Determinants and Policy, Department of Health and Wellbeing, Adelaide, SA, Australia with responsibility for Health in All Policies work. The views expressed in this paper do not necessarily reflect those of the South Australian Government. The other authors have no competing interests.

## Authors’ contributions

AL wrote and revised the paper, contributed to conceptual design of the study and the collection, analysis and interpretation of data. FB led the conceptual design of the study, contributed to the collection, analysis and interpretation of data, and provided comment on drafts. TD contributed to the conceptual design of the study, collection, analysis and interpretation of data, provided comment on drafts. CMcD contributed to conceptual design of the study, the collection, analysis and interpretation of data, and provided comment on drafts. CW contributed to conceptual design of the study and interpretation of data and provided comment on drafts. DMcD contributed to conceptual design of the study and the collection, analysis and interpretation of data and provided comment on drafts. HvE contributed to the analysis and interpretation of data, and provided comment on drafts. All authors read and approved the final manuscript.

## Authors’ affiliations

^1^College of Nursing and Health Sciences, Flinders University, Adelaide, SA, Australia. ^2^Southgate Institute for Health Society and Equity, Flinders University, Adelaide, SA, Australia. ^3^Discipline of Public Health, Flinders University, Adelaide, SA, Australia. ^4^SA Health, Department of Health and Wellbeing, Adelaide, SA, Australia. ^5^The Poche Centre for Indigenous Health and Well-being, Flinders University, Adelaide, SA, Australia.
